# Emergency department–initiated oral naltrexone for patients with moderate to severe alcohol use disorder: A pilot feasibility study

**DOI:** 10.1111/acem.15059

**Published:** 2025-01-08

**Authors:** Ethan Cowan, Clare O'Brien‐Lambert, Erick Eiting, Edward Bull, Jacqueline Ryder, Yvette Calderon, Edwin Salsitz

**Affiliations:** ^1^ Department of Emergency Medicine Icahn School of Medicine at Mount Sinai New York New York USA; ^2^ Department of Emergency Medicine Rutgers New Jersey Medical School Newark New Jersey USA; ^3^ Department of Psychiatry Icahn School of Medicine at Mount Sinai New York New York USA

**Keywords:** alcohol use disorder, emergency service, hospital, medication‐assisted treatment, naltrexone

## Abstract

**Objectives:**

Alcohol use disorder (AUD) is the most common substance use disorder in the United States. Despite availability of four FDA‐approved medications, fewer than 10% of patients are prescribed medication. This study aimed to evaluate the impact and feasibility of emergency department (ED)‐initiated oral naltrexone in patients with moderate to severe AUD.

**Methods:**

This was a prospective, single‐arm, open‐label, nonrandomized clinical trial conducted a single ED. Consenting participants were adults with moderate to severe AUD who were provided a single 50‐mg dose of oral naltrexone, a 14‐day starter pack of naltrexone, and referral for treatment. Follow‐up was conducted at 14 and 30 days post–ED visit. The primary outcome was engagement in formal addiction treatment. Secondary outcomes included alcohol consumption, craving, quality‐of‐life measures, satisfaction, and safety.

**Results:**

Of 761 patients screened, 21 enrolled and received at least one dose of naltrexone. At 14 days, 29% were engaged in treatment, increasing to 33% at 30 days. There was a decrease in the mean (±SD) number of drinks per day from 5.20 (±4.67) at baseline to 2.23 (±4.35) during the follow‐up period (*p* = 0.078). There was a decrease in alcohol craving scores, with median scores dropping from 19 at baseline to 8.27 during the follow‐up period (*p* < 0.001). Quality‐of‐life measures improved, with a statistically significant increase in the reported number of healthy days (*p* = 0.006) and decrease in depressive symptoms (*p* < 0.001). Reported side effects were mild and satisfaction with the screening process was high.

**Conclusions:**

ED‐initiated oral naltrexone is feasible and acceptable for patients with moderate to severe AUD. While engagement in treatment was moderate, significant reductions in alcohol craving and improvements in quality of life suggest potential benefits. Further research is warranted to confirm these findings.

## BACKGROUND

Alcohol is widely consumed globally, but its overuse contributes to enormous public health, physical, and emotional cost. The World Health Organization (WHO) reported that over 2.3 billion adults used alcohol in the past 12 months, with annual consumption increasing over the past decade.^1^ Alcohol is a leading cause of death and disability worldwide, largely attributable to acute injury, hepatic toxicity, cardiovascular disease, infectious disease, and malignancy.^1^, ^2^ Within the United States, the scope of alcohol use is high; the Substance Abuse and Mental Health Services Administration (SAMHSA) reported that over half of Americans drank in the past 30 days, of whom 45.1% binge drank.^3^ Furthermore, 29.5 million Americans, or 10.6% of those over the age of 12, met the Diagnostic and Statistical Manual of Mental Disorders (DSM‐5) criteria for alcohol use disorder (AUD). The burden of AUD shows marked disparities, with those of Native American, Native Hawaiian, Pacific Islander, and mixed racial backgrounds experiencing rates of up to 15.6%.^3^ In addition, the financial cost of alcohol use is estimated to be over $250 billion in lost productivity and health care costs, and this number is rising.^4^, ^5^

Despite the high prevalence of AUD, the use of Federal Drug Administration (FDA)‐approved evidence‐based medications for AUD remains underutilized. Medications for AUD are widely available in the form of first‐line treatments naltrexone and acamprosate as well as alternative agents such as disulfiram, topiramate, and gabapentin.^6^, ^7^, ^8^, ^9^, ^10^ Of these, naltrexone is the most widely used and has been shown to decrease drinking among patients with minimal adverse effects.^11^, ^12^ Despite the proven benefit of naltrexone therapy, even among patients admitted to substance use disorder treatment facilities, the number started on treatment ranges between 0.5% and 1.6%.^13^ The number is similarly low for the broader population of Americans with AUD, of whom only 0.9% receive some form of medication‐assisted treatment (MAT).^3^

The widespread underutilization of medications for AUD, despite overwhelming evidence of efficacy, provides ample opportunity to initiate these medications in nontraditional settings. One potential setting is the emergency department (ED) where large volumes of patients present with intersecting vulnerabilities, such as a lack of access to primary care and outpatient treatment.^14^, ^15^ Thus, treating AUD within the ED has the potential to reach a large number of patients without access to traditional outpatient addiction medicine services.^16^, ^17^ AUD is particularly prevalent in the ED setting, where it contributes to an average of 2 million visits annually.^18^ The annual prevalence of AUD or substance use disorder among U.S. ED patients rose by 30% between 2014 and 2018 totaling 9.4% of all ED visits.^19^ Prior studies have shown that ED‐initiated medication is a viable modality for therapy initiation.^20^, ^21^ However, these studies have analyzed small cohorts in specific urban populations, assayed varying formulations of naltrexone, reported limited outcomes, and are few in number. Thus, it is important for additional research to further characterize the viability, effects, and limitations of ED‐initiated naltrexone therapy.

In this study we aimed to evaluate the impact of immediate ED‐initiated oral naltrexone in patients with moderate to severe AUD on the primary outcome of engagement in formal addiction treatment at 14 and 30 days after the ED visit. We also sought to assess the feasibility of conducting a future large randomized clinical trial in this setting.

## MATERIALS AND METHODS

### Study setting and population

This was a prospective single‐arm, open‐label, nonrandomized clinical trial in patients with moderate to severe AUD presenting to the Mount Sinai Beth Israel ED between July 2021 and October 2023. The Mount Sinai Beth Israel ED is an urban community hospital with an annual census of approximately 75,000 adult visits. Screening for substance use disorder in ED settings is mandated by public health law (Part 405.9 of Title 10). TREND Reporting Guidelines for Nonrandomized/Quasi‐Experimental Study Designs was used to guide reporting of study methodology and results.^22^

### Screening and recruitment

Screening for study enrollment was performed by trained clinical research coordinators (CRCs). During study hours the CRC would review the ED tracking board to identify patients with chief complaints that might indicate eligibility. This could include patients presenting to the ED for complaints related to alcohol use including intoxication or seeking alcohol use treatment or detoxification. If a patient was deemed potentially eligible based on tracking board review, the CRC would approach the patient's ED provider to ascertain if the patient was medically and psychiatrically stable to be approached for potential study participation.

Study staff would then approach the patient and obtain verbal consent to complete a set of screening assessments that includes questions about alcohol use embedded in a general health screener that also includes questions about safety, tobacco, and opioid use in the past 30 days.^23^ If alcohol use was reported in response to the general health screener, the CRC performed a structured diagnostic interview with questions based on the DSM‐5 criteria to evaluate for the presence of moderate/severe AUD and administered the Drug Abuse Screening Test (DAST‐10).^24^, ^25^ Patients not meeting criteria for moderate/severe AUD were given referrals as per existing ED protocols. Patients meeting criteria for moderate/severe AUD were asked to sign informed consent for the study and provide a urine sample to check for opioids. If the urine test did not contain any opioid the patient was offered participation in the full study. Eligible patients had to be 18 years of age or older, able to speak and understand English, and have one point of contact available (in addition to their own) for follow‐up after ED discharge.

Patients who were initially too intoxicated to consent for study participation were reassessed for sobriety and ability to consent by the CRC throughout the course of their ED stay. Patients displaying symptoms of alcohol withdrawal were treated according to a standardized ED protocol and were offered admission to a medically managed detoxification program or hospital, depending on the severity of their withdrawal symptoms. Patients with a history of withdrawal who did not wish to undergo medically managed detoxification but were still interested in reducing their alcohol intake were eligible for study participation.

Patients were excluded from study participation if they (1) had a history of self‐reported opioid use disorder or scored greater than zero on the DAST‐10 where the primary drug reported was an opioid, (2) had a present or anticipated need for opioid medications for pain, (3) were being admitted for alcohol detoxification or other medical and/psychiatric reasons, (4) were too intoxicated to consent, or (5) were pregnant or breastfeeding. Patients were also excluded if they had cirrhosis by past medical history or self‐report because FDA labeling of oral naltrexone indicates that hepatic impairment can significantly alter naltrexone bioavailability and dose‐related hepatotoxicity.

### Baseline visit

Participants who signed consent and met all inclusion criteria and with no exclusion criteria were provided a single dose of 50 mg of oral naltrexone and observed for 60 minutes to assess for any medication side effects or adverse events. During this observation period patients completed the following assessments; (1) stigma scale,^26^ (2) ASSIST‐Lite,^27^ (3) cannabis assessment, (4) health status report, (5) crime and justice assessment, (6) daily substance use report using timeline follow‐back methodology,^28^ (7) health services utilization report,^29^ (8) health‐related quality of life (HRQoL),^30^ (9) alcohol craving scale,^31^ (10) generalized anxiety disorder screen (GAD‐7),^32^ (11) patient health questionnaire (PHQ‐9),^33^ and (12) a satisfaction survey. Patients who did not have liver function testing (LFT) in the 30 days prior to enrollment were asked to undergo phlebotomy to assess liver enzymes. LFT results were not required prior to ED discharge. After the 60‐min period of observation participants were given a 14‐day starter pack of 50‐mg oral naltrexone tablets and instructed to take one tablet daily. The CRC also provided the participant with information on the hospital's outpatient addiction program that had daily open‐access walk‐in hours 7 days a week. After the patient was discharged from the ED, the CRC sent a message to the addiction medicine program informing them that the patient was seen in the ED, enrolled in the study, and started on oral naltrexone, thus providing a warm handoff to follow‐up care. Patients found to have LFTs greater than 5× the upper limit of normal were discontinued from the study for safety reasons and not provided with the naltrexone starter pack or, if already discharged from the ED, were contacted and told to stop taking oral naltrexone.

### Follow‐up visits and assessments

On a daily basis (excluding the 14‐ and 30‐day follow‐up assessment visits) participants were asked to complete a craving scale and daily substance use electronic report using Qualtrics. For patients without a device capable of receiving and completing electronic surveys these daily assessments were performed over the phone. At 14 and 30 days post–ED visit participants were contacted via phone and asked to complete a (1) daily substance use report (timeline follow‐back methodology),^28^ (2) ASSIST‐Lite,^27^ (3) HRQoL,^30^ (4) medication adherence,^34^ (5) Patient Related Inventory of Side Effects (PRISE),^35^ (6) health services utilization report, (7) GAD‐7,^32^ and (8) PHQ‐9.^33^ All patient reports of engagement in treatment at 14 and 30 days were independently verified by direct contact with the treating program/provider and the American Society of Addiction Medicine level of care recorded.^36^ Protocolized adverse event and protocol deviation reporting were employed throughout the study period in accordance with good clinical practice guidelines.^37^ Participants could be compensated up to $290 for completing all study visits and daily assessments.

### Outcomes

The primary outcomes were engagement in comprehensive addiction treatment at 14 and 30 days post‐enrollment. Secondary outcomes included descriptive assessments of stigma, medication side effects, other substance use, satisfaction, adverse events, and serious adverse events. Secondary outcomes for alcohol consumption, alcohol craving, health care utilization, depression/anxiety, medication adherence, and HRQoL were assessed for changes between baseline and 14‐ and 30‐day follow‐up. A full statistical plan for secondary outcomes not specifically discussed below can be found in the online supplement.

### Statistical analysis and power

In general, statistical analysis was performed using Stata 15.1 (StatCorp, LLC). Descriptive statistics (frequencies, measures of central tendency, and dispersion) were computed to characterize the data. Results were considered statistically significant at *p* < 0.05. Missing data were not imputed. All comparisons were preplanned and described on clinicaltrials.gov (NCT04817410).

### Primary and secondary outcome assessment

Given the lack of a control group, the primary outcome of engagement in comprehensive addiction treatment is descriptively reported as counts. Participants lost to follow‐up were assumed to not be engaged in addiction treatment.

### Alcohol intake and craving

At baseline, the total number of drinks consumed during each of the timeline follow‐back days 1–7 for each participant divided by 7 days was operationalized as the average number of drinks consumed in a 24‐h period (i.e., per day) at baseline for each participant. Likewise, throughout the 30‐day follow‐up period, participants reported totals for beer, wine, and liquor consumed daily. The total number of drinks consumed per day by each participant during the entire follow‐up period divided by the respective number of days for which the participant provided a response was operationalized as the average number of drinks consumed per day during the follow‐up period. The comparison of the baseline to follow‐up average number of drinks consumed per day was assessed using the Wilcoxon signed‐rank test for related groups (each individual paired with their own data). Participants who did not complete any follow‐up assessments were excluded from the paired analysis. The number of heavy drinking days, defined as four or more drinks for women and five or more drinks for men on the same occasion in a 24‐h period was also calculated. We also calculated the number of days of self‐reported zero alcohol intake and zero alcohol craving. For both the baseline and the follow‐up periods, zero drinks per day was only indicated if the participant explicitly indicated that they did not drink within the 24‐h period for which they were reporting.

Participants also completed the Penn Alcohol Craving Scale (PACS) during the baseline and follow‐up periods.^30^ At enrollment, participants were asked the PACS survey questions with respect to the last week that they drank any alcohol. An overall craving score was then calculated for each participant for the baseline period by summing their scores for each survey question. Participants then completed daily PACS via Qualtrics. To calculate the average craving score for each participant, daily craving scores completed over the course of the 30‐day follow‐up period were totaled for each participant and divided by the respective number of days for which the participant provided a response. For both the baseline and the follow‐up periods, incomplete survey responses on PACS were not given a score. The comparison of the baseline to follow‐up average daily craving was assessed using the Wilcoxon signed‐rank test for related groups (each individual paired with their own data). Participants who did not complete any follow‐up assessments were excluded from the paired analysis.

### Sample size

Given that the purpose of this pilot study was to demonstrate the feasibility of ED‐initiated MAT for AUD, the sample size was based on the projected ability to recruit participants over a 12‐month period. Given our previous experience recruiting for a prior ED initiated treatment trial for opioid use disorder we anticipated being able to recruit two participants per month with an expected attrition rate of 15%.

## RESULTS

### Participant population demographic characteristics

**FIGURE 1:**
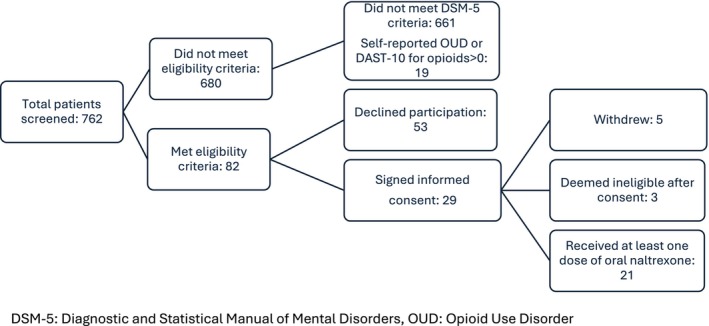
Participant flow diagram. DSM‐5, Diagnostic and Statistical Manual of Mental Disorders; OUD, opioid use disorder.

Between July 2021 and October 2023, a total of 762 patients were screened for study enrollment (Figure 1). Of these, 680 did not meet inclusion criteria. Of the 82 who met inclusion criteria, 29 participants signed informed consent for participation and provided a urine drug screen, of which none contained opioids. Of the 29 patients who signed consent, five withdrew from the study prior to receiving naltrexone. One eloped from the ED and four said they no longer wished to participate without providing a reason. Three additional participants were withdrawn from the study by the principal investigator prior to receiving oral naltrexone. One had been enrolled due to a protocol deviation due to missed exclusion criteria. One participant had elevated liver function tests identified during their ED visit and another was administered morphine for pain. In total, 21 participants received at least one dose of oral naltrexone. The baseline characteristics of participants who received at least one dose of oral naltrexone (hereto forth referred to as “enrolled participants”) is shown in Table 1. The population was predominantly male (81%). Median age was 41 years. More than half of enrolled participants identified as Latino/Hispanic. Caucasians comprised the most common racial group at 43%. One‐third of the participants were either unemployed or disabled at the time of study entry, most (86%) indicated active health insurance coverage, and 43% expressed concern about their housing situation over the next 2 months.

**TABLE 1:** Enrolled participant characteristics.

Subgroup	Number (%)^a^
Age (years), median (IQR)	41 (30–55)
Gender	
Male	17 (81)
Female	4 (19)
Ethnicity	
Hispanic/Latino	11 (52)
Not Hispanic/Latino	9 (43)
Missing	1 (5)
Race	
Black or African American	4 (19)
White	9 (43)
More than one race	2 (10)
Unknown/not reported	6 (29)
Highest level of education	
Some high school, no diploma	4 (19)
High school graduate/GED or equivalent	3 (14)
Technical/trade/vocational school	2 (10)
Junior (2 year) college	1 (5)
Some college (4‐year college or university)	5 (24)
College graduate (4‐year college or university)	3 (14)
Postcollege/graduate degree	1 (5)
Missing	1 (5)
Estimated total income	
Less than $1000	2 (10)
$10,001–$20,000	1 (5)
$20,000–$40,000	3 (14)
$40,000 or more	8 (38)
Don't know/missing	7 (33)
Past‐year employment status	
Unemployed	4 (19)
Employed	7 (33)
Disabled, permanently or temporarily	3 (14)
Other	7 (33)
Covered by health insurance/health plan	18 (86)
Concerned about stable housing over the next 2 months	9 (43)
Incarcerated in the past 12 months	0 (0)

^a^ Not all categories add to 100% due to rounding.

### Primary outcome

**FIGURE 2:**
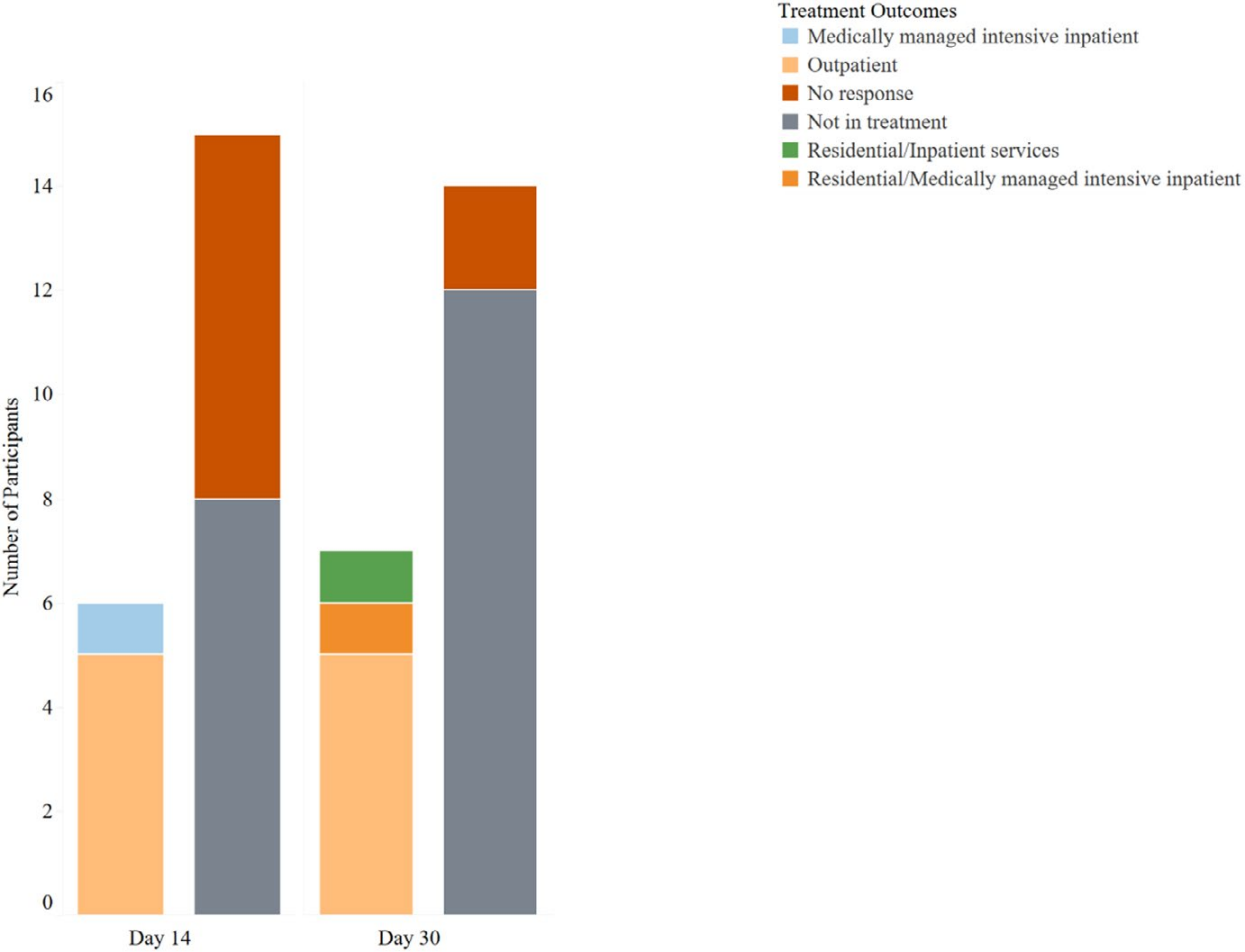
Primary outcome: engagement in addiction treatment. Number of participants in treatment at Days 14 and 30.

The primary outcome, engagement in comprehensive addiction treatment at 14 and 30 days post‐enrollment, is shown in Figure 2. At 14 days post–ED‐initiated oral naltrexone, six (29%) were in treatment, mostly outpatient care. At 30 days, seven (33%) were in treatment, again primarily outpatient care. Of the six engaged in treatment at 14 days, three were still in treatment on Day 30. Of the 15 not engaged in treatment at 14 days, four were engaged in treatment at 30 days. For the seven participants engaged in treatment at 30 days, two indicated that they were continuing oral naltrexone, three had decided to stop naltrexone, and two were missing treatment information.

### Secondary outcomes

#### Alcohol consumption and cravings

**FIGURE 3:**
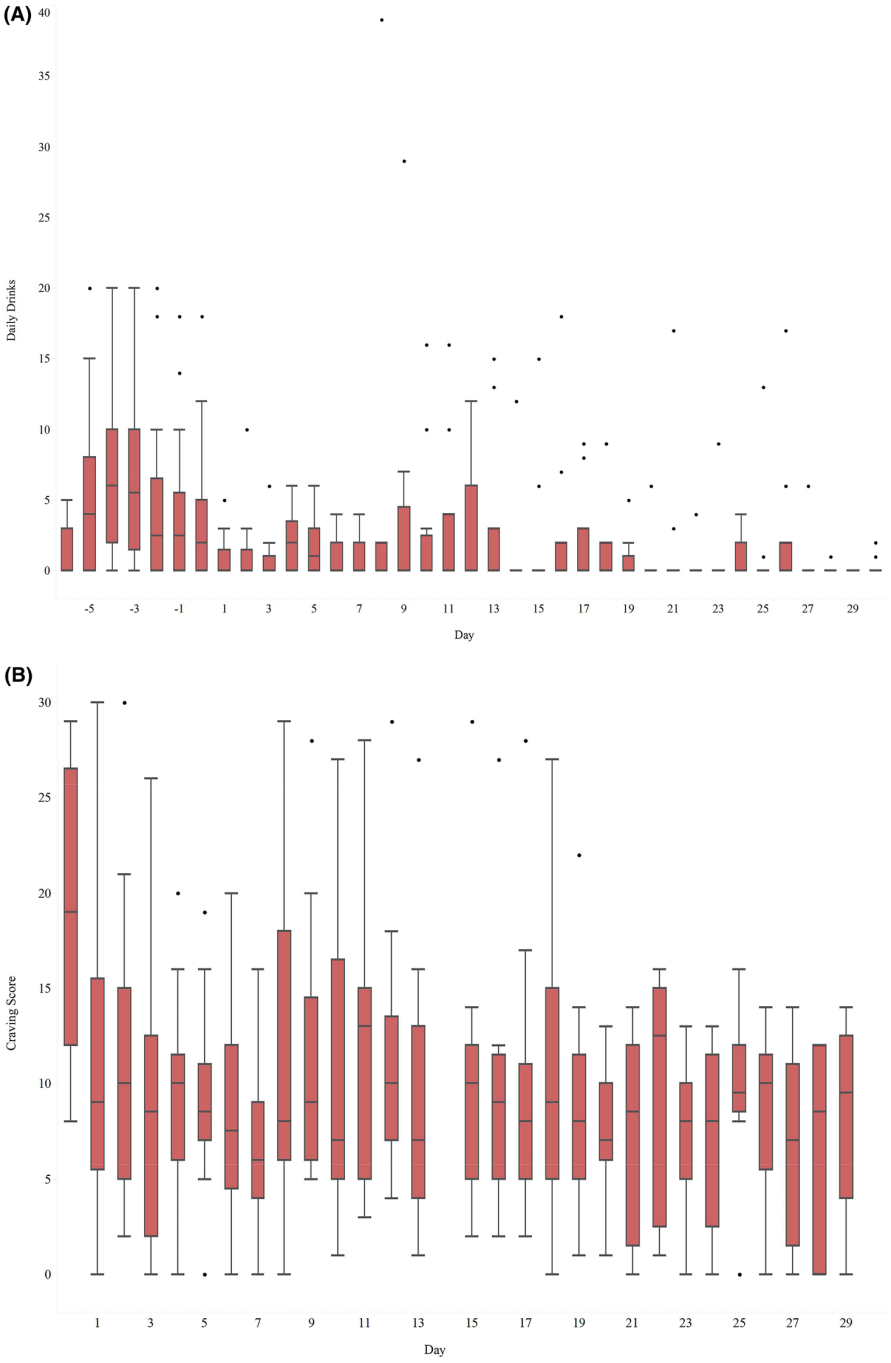
Participant self‐reported alcohol intake (A) and alcohol craving (B) over 30 days. (A) Box‐and‐whisker plot of self‐reported number of alcoholic drinks consumed per day. Each colored box displays the median self‐reported daily alcohol intake and interquartile range (IQR), with whiskers extending to the furthest data point within 1.5 times the IQR and outliers shown as individual data points. (B) Participant self‐reported daily alcohol craving over 30 days. Each colored box displays the median self‐reported daily alcohol craving score and IQR, with whiskers extending to the furthest data point within 1.5 times the IQR and outliers shown as individual data points.

Figure 3 depicts the cravings and alcohol intake for participants over the 30‐day study period. The median (IQR) overall number of drinks consumed per day was 4.29 (2.29–7.57) and 0.73 (–2.81) during the baseline and follow‐up periods, respectively. When comparing among study participants, there was no significant difference observed between the average number of drinks consumed per day during baseline and the follow‐up period using the Wilcoxon signed‐rank test (*Z* = 1.79, *p* = 0.078). The median (IQR) overall daily craving score obtained was 19 (12–26.5) at baseline and 8.27 (4.65–11.75) during the follow‐up period where 0 represents no craving and 36 represents the most severe craving.^30^ An exact sign test was used to compare overall alcohol craving scores among study participants and showed a significant median decrease in the overall alcohol craving score by 11.5 examining the follow‐up period compared to baseline (*p* < 0.01).

#### Quality of life measures

There was no significant difference observed when examining the distribution of raw GAD‐7 scores attained at baseline and Day 30 (Table S8). There was a significant difference observed when examining the distribution of raw PHQ‐9 scores attained at baseline and Day 30, with higher overall depression reported at baseline (median of 9—mild depression) compared to Day 30 (4—minimal depression; Wilcoxon signed rank *Z* = 3.20, *p* < 0.001; Table S7). On the HRQoL, there was a statistically significant difference in the number of unhealthy days reported at baseline compared to Day 30, with fewer unhealthy days reported overall at Day 30 (18 unhealthy days) compared to baseline (20 unhealthy days; Wilcoxon signed‐rank *Z* = 2.91, *p* = 0.002; Table S6).

#### Participant satisfaction

Participant convenience and satisfaction with our screening process for ED‐initiated oral naltrexone was high (Table 2) with 67% reporting that the process was not inconvenient, 62% reporting that the process was not difficult, and 74% reporting that they were very satisfied with the screening process.

**TABLE 2:** Baseline convenience and satisfaction with ED screening process.

Convenience of screening at baseline						
Numbers selected on a scale from 0 to 6 where 0 = not inconvenient and 6 = very inconvenient						
0	1	2	3	4	5	6
66%	4%	4%	9%	0%	4%	8%

Difficulty of screening at baseline						
Numbers selected on a scale from 0 to 6 where 0 = not at all difficult and 6 = very difficult						
0	1	2	3	4	5	6
62%	14%	9%	0%	0%	4%	9%

Satisfaction of screening at baseline						
Numbers selected on a scale from 0 to 6 where 0 = very dissatisfied and 6 = very satisfied						
0	1	2	3	4	5	6
5%	5%	5%	4%	5%	5%	76%

#### Safety events

As assessed by the PRISE questionnaire,^34^ reported side effects were not uncommon but were generally mild. At Day 14, many participants (10) indicated some form of gastrointestinal that included diarrhea (four), constipation (four), dry mouth (four), and nausea/vomiting (six). At Day 30, gastrointestinal and sleep symptoms were again the most common category, with seven participants reporting symptoms of diarrhea (two), constipation (two), dry mouth (five), nausea/vomiting (two), difficulty sleeping (six), and sleeping too much (three). Eight (eight) participants also reported other symptoms including anxiety (six), poor concentration (four), general malaise (two), restlessness (five), fatigue (four), decreased energy (five), and muscle weakness (one; Table S2). There were no serious adverse events attributable to oral naltrexone.

### Other secondary outcomes

Medication adherence was good to excellent in 71% and 80% of patients at 14 and 30 days, respectively (Table S4). There was no change in health care utilization or HRQoL from baseline to 30 days (Table S5) and no participants transitioned to long‐acting injectable naltrexone during the follow‐up period. Baseline participant reported stigma was mixed. Most participants (80%) strongly agreed that people would not want to marry someone with an alcohol problem, believe people look down upon (66%), or think less of (67%) those hospitalized for an alcohol problem and that people are afraid of people with an alcohol problem (57%). Responses to the other stigma questions were generally evenly split (Table S1). After alcohol, at baseline, the next most reported used substances on the ASSIST‐Light survey were tobacco (57% people used in past 3 months) and then cannabis (48%; Table S3).

## DISCUSSION

Despite recent recommendations^38^ for ED providers to identify AUD and initiation medication treatment, a recent systematic review performed as part of the Society for Academic Emergency Medicines (SAEM) Guidelines for Reasonable and Appropriate Care in the Emergency Department (GRACE) concluded that, “there is relatively little evidence addressing the initiation of anticraving medications for AUD in the ED.”^39^ This prospective, single‐arm, open‐label, nonrandomized clinical trial adds to the limited literature on ED‐initiated medications for AUD and adds several important findings to the literature.

The primary outcome, engagement in comprehensive addiction care at 14 and 30 days post–ED‐initiated oral naltrexone, was better than in earlier studies. The proportion of participants engaged in care at 14 days was 29% at 14 days and 33% at 30 days. This proportion is higher than reported by Anderson et al.^20^ in a study of ED‐initiated oral and injectable naltrexone where the proportion of participants attending an addiction medicine follow‐up was 9.1% for those prescribed oral naltrexone and 27.8% for those receiving long‐acting injectable naltrexone. This discrepancy could be attributed to differences in study design, as their intervention did not include daily surveys or multiple follow‐ups but rather a prescription for naltrexone alongside a single follow‐up at a bridge clinic. Conversely, in another open‐label single‐arm clinical trial examining a series of long‐acting injectable naltrexone, Murphy et al.^21^ found that 84% of participants attended their 4‐week follow‐up visit. There are several possible reasons for these observed differences between studies beyond simple differences in population characteristics. Our study involved daily interactions with participants through daily surveys, which may have kept participants more engaged. We also have an on‐site comprehensive addiction treatment program that likely made it easier for patients to make follow‐up appointments. While we did provide a follow‐up incentive for participants that could have inflated our engagement numbers, the incentive was only given for completing survey measures and was not tied to follow‐up care.

We also provide data on several important secondary outcomes including alcohol craving and daily alcohol intake. In this study, we found a statistically significant difference in daily cravings but no change in overall alcohol use. This is in sharp contrast to patients receiving injectable naltrexone in the study by Murphy et al.^21^ where they observed a decrease of 7.5 drinks per day. Some of this difference may be due to the assessment of alcohol use and the use of an injectable versus oral form of naltrexone. While both studies use self‐reported outcomes and timeline follow‐back methodology, we collected data on alcohol intake daily, using a web‐based survey, which may have led to increased accuracy of reported drinking behavior. Furthermore, while adherence to oral naltrexone was reported as good to excellent in 71% and 80% of patients at 14 and 30 days, respectively, we did not perform formal pill counts to confirm compliance.

While we did not observe a decrease in average daily alcohol consumption, participants did report a significant decrease in average daily alcohol craving. To our knowledge, this is the only ED‐based clinical trial to measure alcohol craving after discharge in a systematic way. While one could argue that a reduction in alcohol craving without a reduction in alcohol intake would not have an impact on alcohol‐related morbidity it is possible that reduced cravings could improve overall quality of life and potentially lead to reductions in alcohol intake over a longer period. While it is not possible to attribute reduced craving to improved patient‐centered outcomes, we did find that study participants had improved quality‐of‐life ratings, reduced anxiety, and reduced depressive symptoms over the study period. There is a strong consensus on the bidirectional effect of alcohol on depression and anxiety, suggesting that improvement of mood disorders could positively influence alcohol use and vice versa.^40^, ^41^, ^42^, ^43^ Given the challenges faced by patients with AUD, even modest shifts in mental health could augment success in management of their alcohol use.

As found in other studies of oral naltrexone, we did not identify any safety concerns in participants taking oral naltrexone nor is there any reason to believe ED patients differ in any physiological way to patients receiving oral naltrexone in other settings that could increase risk.^44^ While our study is too small to identify rare safety events, we did not identify any serious adverse events related to oral naltrexone and participant‐reported side effects tended to be mild. Of note, one patient was withdrawn from the study due to elevated LFTs. While recent literature has challenged the warnings of oral naltrexone in patients with significant liver disease, we chose to be conservative and exclude those with aspartate transaminase and alanine aminotransferase elevations 5× normal.^45^

Other measured secondary outcomes are more challenging to interpret. The population's baseline health care utilization was low and did not change over the study period. This was unexpected given the fact that patients with AUD have high rates of ED visits and admissions.^19^ Participant‐reported responses to the baseline stigma questionnaire are interesting and warrant further study. Their relatively high self‐perceived stigma toward people with AUD could impact their engagement in follow‐up care as previously demonstrated in large epidemiological studies.^46^ A strong association between self‐perceived stigma and engagement in care could indicate a need to integrate sigma reducing interventions into ED‐based care for patients with AUD.

Regarding the significance of this feasibility study, it is valuable to first characterize the current landscape of ED‐initiated AUD medical therapy. Despite the increasing rate of AUD among ED patients, less than 15% of EDs have formal screening and intervention policies in place for AUD.^47^ The reported barriers to implementing ED‐initiated naltrexone programs include factors such as high staff turnover, insufficient training, and high patient acuity, though there are resources available for mitigating these challenges.^48^, ^49^ Regarding the viability of our screening protocol, 62% of our patients found the screening to not be difficult, 67% found it to be convenient, and 74% were satisfied with the screening. Given the available resources for training ED staff and the subjective patient assessment of the screening as convenient, there is potential for more EDs to implement similar approaches. Regarding the long‐term benefits of such training, one study saw that staff training increased naltrexone prescription rates from 0.66% to 3.96% among patients with AUD over 2 years.^50^ As a whole, both our study and those described show the potential viability of ED‐initiated naltrexone, but significant gaps in our understanding of the implementation barriers remain and must be addressed by further study.

## LIMITATIONS

This study had several limitations. Due to the nonrandomized design, lack of a nontreatment control and small sample size, drawing inferences about efficacy of ED‐initiated naltrexone on short‐term engagement in care is not possible. Our purpose, however, was to demonstrate acceptance and feasibility for planning a future multicenter randomized controlled trial, which we believe we have done. Furthermore, while steps were taken to reduce enrollment bias, convenience sampling, nonblinded research coordinators, and the self‐selection of patients motivated to change their alcohol usage could have introduced enrollment bias. We also sought out a very narrow cohort for study inclusion by excluding patients being admitted for inpatient medical treatment or medically managed alcohol detoxification, which further limits generalizability. Most EDs, however, do not have access to on‐site inpatient addiction treatment; thus there is likely a larger cohort of individuals who might be eligible and willing to initiate oral naltrexone at other centers, but this requires further study. Lastly, recruitment was heavily impacted by the concurrent COVID‐19 pandemic, which led to limitations of our research staff and a prolonged recruitment period.

## CONCLUSIONS

In this prospective non‐randomized clinical trial, we found that ED‐initiated oral naltrexone for alcohol use disorder is acceptable to patients and feasible. Engagement in comprehensive addiction care among this cohort at 14 and 30 days was moderate compared to similar studies. While there was no significant reduction in the average number of daily drinks consumed, there was a significant decrease in alcohol cravings and improvement in quality‐of‐life measures. Further study of ED‐initiated naltrexone is warranted.

## FUNDING INFORMATION

This work was supported in part through the computational and data resources and staff expertise provided by Scientific Computing and Data at the Icahn School of Medicine at Mount Sinai and supported by the Clinical and Translational Science Awards (CTSA) grant UL1TR004419 from the National Center for Advancing Translational Science. The work was directly supported by a grant from the Icahn School of Medicine at Mount Sinai Addiction Research Center.

## CONFLICT OF INTEREST STATEMENT

The authors declare no conflicts of interest.

## Supporting information


Data S1.

## Data Availability

The data that support the findings of this study are available from the corresponding author upon reasonable request.
