# Supplementary material for: Emergency department–initiated oral naltrexone for patients with moderate to severe alcohol use disorder: A pilot feasibility study
Source: Acad Emerg Med. 2025 Jan 8;32(5):488–97. doi: 10.1111/acem.15059 (PMC12077059; doi:10.1111/acem.15059)
Supplement: Supplementary file 1 — Data S1. [file ACEM-32-488-s001.pdf]

Supplemental Table A: Baseline participant reported stigma

Subgroup	Strongly Agree (N(%))	Agree (N(%))	Disagree (N(%))	Strongly Disagree (N(%))
Most people believe that people who formerly had an alcohol problem cannot be trusted	3 (14)	7 (33)	6 (29)	5 (24)
Most people would not want to marry someone who has had an alcohol problem*	2 (10)	12 (60)	3 (15)	3 (15)
Most people believe that a person who has been hospitalized for an alcohol problem is dangerous	2 (10)	8 (38)	8 (38)	3 (14)
Most people will think less of a person who has been hospitalized for an alcohol problem	2 (10)	12 (57)	5 (24)	2 (10)
Most people look down on people who have been hospitalized for an alcohol problem	3 (14)	11 (52)	6 (29)	1 (5)
Most people think that patients with an alcohol problem are just as intelligent as the average person	3 (14)	12 (57)	6 (29)	0 (0)
Most employers will not hire a person who has been hospitalized for an alcohol problem	2 (10)	8 (38)	10 (48)	1 (5)
Many people are afraid of people who have been patients for an alcohol problem	3 (14)	9 (43)	8 (38)	1 (5)

*One participant did not answer this question. No all % add to 100% due to rounding

For Review Only

Supplemental Table B: Patient Rated Inventory of Side Effects (PRISE)

Symptoms experienced in past week by those started on oral naltrexone	Day 14		Day 30	
Organ System	Yes (N (%))	No (N (%))	Yes (N (%))	No (N (%))
GASTROINTESTINAL	10 (71)	4 (29)	7 (47)	8 (53)
HEART	5 (36)	9 (64)	4 (27)	11 (73)
SKIN	6 (43)	8 (57)	2 (13)	13 (87)
NERVOUS SYSTEM	6 (43)	8 (57)	4 (27)	11 (73)
EYES/EARS	4 (29)	10 (71)	3 (20)	12 (80)
GENITAL/URINARY	2 (14)	12 (86)	6 (40)	9 (6)
SLEEP	9 (64)	5 (36)	7 (47)	8 (53)
SEXUAL FUNCTIONING	1 (7)	13 (93)	4 (27)	11 (73)
OTHER	8 (57)	6 (43)	8 (53)	7 (47)

For Review Only

Supplemental Table C: Alcohol, Smoking and Substance Involvement Screening Tool – Lite

ASSIST-Lite	Baseline		Day 14		Day 30	
	Yes (N (%))	No (N (%))	Yes (N (%))	No (N (%))	Yes (N (%))	No (N (%))
Did you smoke a cigarette containing tobacco?	12 (57)	9 (43)	6 (40)	9 (60)	8 (42)	11 (58)
Did you usually smoke more than 10 cigarettes each day?	4 (33)	8 (67)	2 (33)	4 (67)	3 (38)	5 (63)
Did you usually smoke within 30 minutes after waking?	7 (58)	5 (42)	1 (17)	5 (83)	3 (38)	5 (63)
Did you have a drink containing alcohol?	21 (100)	0 (0)	10 (67)	5 (33)	14 (74)	5 (26)
On any occasion, did you drink more than 4 standard drinks of alcohol?	20 (95)	1 (5)	6 (60)	4 (40)	3 (21)	11 (79)
Have you tried and failed to control, cut down, or stop drinking?	19 (90)	2 (10)	5 (50)	4 (40)	7 (50)	7 (50)
Has anyone expressed concern about your drinking?	18 (86)	3 (14)	3 (30)	7 (70)	5 (36)	9 (64)
Did you use cannabis?	10 (48)	11 (52)	1 (7)	14 (93)	5 (26)	14 (74)
Have you had a strong desire or urge to use cannabis at least once a week or more often?	3 (30)	7 (70)	0 (0)	1 (100)	2 (40)	3 (60)
Has anyone expressed concern about your use of cannabis?	1 (10)	9 (90)	0 (0)	1 (100)	1 (20)	4 (80)
Did you use an amphetamine-type stimulant, or cocaine, or a stimulant medication not as prescribed?	7 (33)	14 (67)	2 (13)	13 (87)	0 (0)	18 (95)
Did you use a stimulant at least once each week or more often?	1 (14)	6 (86)	2 (100)	0 (0)	0 (0)	0 (0)
Has anyone expressed concern about your use of stimulant?	5 (71)	2 (29)	0 (0)	2 (100)	0 (0)	0 (0)
Did you use a sedative or sleeping medication not as prescribed?	3 (14)	18 (86)	2 (13)	13 (87)	0 (0)	19 (100)
Have you had a strong desire or urge to use a sedative or sleeping medication at least once a week or more often?	2 (67)	1 (33)	1 (50)	1 (50)	0 (0)	0 (0)
Has anyone expressed concern about your use of sedative or sleeping medication?	1 (33)	2 (67)	1 (50)	1 (50)	0 (0)	0 (0)
Did you use a street opioid (e.g. heroin) or an opioid-containing medication not as prescribed?	0 (0)	21 (100)	0 (0)	15 (100)	0 (0)	19 (100)
Have you tried and failed to control, cut down, or stop using an opioid?	0 (0)	0 (0)	0 (0)	0 (0)	0 (0)	0 (0)
Has anyone expressed concern about your use of an opioid?	0 (0)	0 (0)	0 (0)	0 (0)	0 (0)	0 (0)
Did you use any other psychoactive substances?	1 (5)	20 (95)	1 (7)	14 (93)	0 (0)	19 (100)

*Baseline asked about substance use during past 3 months. Day 14 and 30 asked about past two weeks. Not all percents add up to 100 due to refusals to respond.

For Review Only

Supplemental Table D: Guidelines Regimen Information Program (GRIP)

GRIP Survey Categories	Range of Scores	Time Point	N	Median (IQR)
Very poor = 1<br>Poor = 2<br>Fair = 3<br>Good = 4<br>Very good = 5<br>Excellent = 6	1-6	Day 14	14	4.5 [(3) - (5)]
		Day 30	15	4 [(4) - (5)]

There is no statistically significant difference between day 14 and day 30 responses for adherence to oral naltrexone (Wilcoxon Signed-rank Z= -0.76, p=0.50).

For Review Only

Supplemental Table E: Healthcare Utilization

Visit Type	Baseline N	Baseline Median (IQR)	Day 14 N	Day 14 Median (IQR)	Day 30 N	Day 30 Median (IQR)	Friedman's Chi-Sq	P-value
Inpatient Admissions	21	0 [(0) - (0)]	15	0 [(0) - (0)]	19	0 [(0) - (0)]	6.74	0.915
Outpatient Visits	20	0 [(0) - (1)]	14	0.5 [(0) - (1)]	19	0 [(0) - (1)]	10.85	0.456
Emergency Department Visits	20	0 [(0) - (0)]	15	0 [(0) - (0)]	19	0 [(0) - (0)]	5.03	0.957

Baseline asked about health care visits in the 7-days prior to the baseline enrollment visit. Follow-up health service utilization asked about health care visits in the preceding 14-days. There were no significant differences observed in inpatient admissions, outpatient visits or ED visits between baseline, 14- and 30-days.

For Review Only

Supplemental Table F: Health Related Quality of Life

Self-reported general health	Range of Scores	Time Point	N	Median (IQR)
Excellent = 1<br>Very good = 2	0-5	Baseline	21	3 [(2) - (4)]
Good = 3<br>Fair = 4		Day 14	15	3 [(3) - (4)]
Poor = 5<br>Don't Know/Not Sure = 0		Day 30	19	3 [(2) - (3)]

There is no statistically significant difference between baseline, day 14 and day 30 responses for self-reported general health (Friedman’s chi-sq =20.55, p=0.057)

Unhealthy Days Score	Range of Scores	Time-Point	N	Median (IQR)
Overall Score	0-30	Baseline	20	20.5 [(6) – (30)]
		Day 14	15	22 [(10) – (30)]
		Day 30	18	11.5 [(0) – (30)]

There is a statistically significant difference between baseline and day 30 reported unhealthy days (Wilcoxon Signed-rank Z= 2.91, p= 0.002)

Supplemental Table G: Comparison of PHQ-9 scores at baseline, 14 and 30-days

Depression Severity	Range of Scores	Time Point	N	Median (IQR)
Minimal depression (1-4)<br>Mild depression (5-9)<br>Moderate depression (10-14)<br>Moderately severe depression (15-19)<br>Severe depression (20-27)	0-26	Baseline	21	9 [(4) - (19)]
		Day 14	13	10 [(7) - (15)]
		Day 30	19	4 [(2) - (8)]

There is a statistically significant difference between baseline and 30-day PSQ-9 scores (Wilcoxon signed rank Z = 3.20, p < 0.001) and between 14-day and 30-day PSQ-9 scores (Wilcoxon signed rank Z = 2.56, p = 0.009).

For Review Only

Supplemental Table H: GAD-7 comparison of GAD-7 scores at baseline, 14 and 30 days

Anxiety Severity	Range of Scores	Time Point	N	Median (IQR)
Minimal anxiety (0-4)<br>Mild anxiety (5-9)<br>Moderate anxiety (10-14)<br>Severe anxiety (15-21)	0-2	Baseline	20	6.5 [(3.5) - (14.5)]
		Day 14	14	7.5 [(2) - (16)]
		Day 30	18	3 [(1) - (9)]

There is no statistically significant difference between GAD-7 scores between baseline, day-14 and day-30 (BL vs day 14 Wilcoxon signed rank Z = 0.81 and p = 0.440, BL vs day 30 Z = 1.90 and p = 0.058, day 14 vs day 30 Z = 1.61 and p = 0.115).

For Review Only
